# Applications of Plasma-Activated Liquid in the Medical Field

**DOI:** 10.3390/biomedicines9111700

**Published:** 2021-11-16

**Authors:** Sungryeal Kim, Chul-Ho Kim

**Affiliations:** 1 Department of Otolaryngology, School of Medicine, Ajou University, Suwon 16499, Korea; entksr@aumc.ac.kr; 2 Department of Molecular Science and Technology, Ajou University, Suwon 16499, Korea

**Keywords:** cold plasma, plasma-activated water, plasma-activated liquid

## Abstract

Much progress has been made since plasma was discovered in the early 1900s. The first form of plasma was thermal type, which was limited for medical use due to potential thermal damage on living cells. In the late 1900s, with the development of a nonthermal atmospheric plasma called cold plasma, profound clinical research began and ‘plasma medicine’ became a new area in the academic field. Plasma began to be used mainly for environmental problems, such as water purification and wastewater treatment, and subsequent research on plasma and liquid interaction led to the birth of ‘plasma-activated liquid’ (PAL). PAL is currently used in the fields of environment, food, agriculture, nanoparticle synthesis, analytical chemistry, and sterilization. In the medical field, PAL usage can be expanded for accessing places where direct application of plasma is difficult. In this review, recent studies with PAL will be introduced to inform researchers of the application plan and possibility of PAL in the medical field.

## 1. Introduction

Since Langmuir introduced plasma in 1928 [1], plasma technology has continued to develop and is now being applied in various areas. In the medical field, superficial thermal effects of argon (Ar) plasma were applied to coagulation function in 1977 [2], but other applications were delayed due to thermal effect. In the 1990s, the improvement of plasma technology had enabled the generation of nonthermal plasma, which has led to active research in the medical field. Unlike thermal plasma, the temperature of electrons is higher than that of heavy particles in nonthermal plasma, which is caused by large differences in mass. Due to this phenomenon, nonthermal plasma is in a low gas temperature state (less than 40 °C), the so-called ‘cold’ plasma. Cold plasma has the following typical characteristics: (1) operation at atmospheric pressure; (2) mean electron temperatures sufficient or electron impact dissociation, excitation, and ionization(>1 eV); (3) mean gas temperatures of <40 °C; (4) transfer of heat to the target below a harmful level; (5) current below the level causing Joule heating [3]. These properties suggest the nonlethality of nonthermal plasma to living cells [4,5,6], as confirmed by many studies during the 20th century. Much research for application in the medical field was conducted, and based on the results, Kong et al. reported applications of plasma in numerous medical areas, including hospital hygiene, antifungal treatment, dental care, skin disease, chronic wound, and cosmetics [4]. Recently, commercialized devices with cold plasma technology have been used in the real-world clinical practice of wound care and cosmetics [5].

The range of applications in the medical area is limited due to several characteristics of the direct application of plasma. (1) Permeability: In the common materials process, plasma can directly modify the uppermost surface layer only, which is about 10 nanometers thick [6]. Deep tissues (muscle, bone, and organs) are 1 millimeter to several centimeters deep that direct application of plasma may be insufficient to exert an effect. Although some studies have argued that reactive species can penetrate the stratum corneum, which is the main barrier of the skin that prevents outside molecules from entering the body [7,8,9], and several studies have shown anticancer effects in cancer implanted under the mouse skin [10,11], the depth of transmission is still insufficient and exact mechanisms are unknown. (2) Manipulation: Direct application of plasma is created directly from the generator, so the device must exist where plasma is used. This means that the use of plasma is limited depending on the presence and shape of the device. Additionally, it is impossible to maintain and transform it because it comes directly from the device. These limitations were also considered in other industries, especially environmental technology and agriculture, where PAL has already been used for water treatment and food preservation [12,13].

After Gukin described the interaction between plasma and liquid in 1887, many research studies have shown the availability of PAL in nanomaterial processing, analytical chemistry, and organic wastewater treatment [14]. Moreover, interaction with microorganisms, including bacteria, fungi, and viruses, has been studied, and inhibition mechanism has been found [14,15]. Based on the results of the effect of PAL on living cells using microorganisms, studies regarding the interaction between PAL and mammalian cell has been actively conducted [16].

In this review, we will discuss the brief mechanism and generation of reactive species in PAL and its applications in medicine.

## 2. Electric Discharge and Reactive Species

The mechanism of action of cold plasma involves reactive particles, which comprise reactive species, radiation (ultraviolet, visible light, near-infrared radiation), and electromagnetic fields [3]. Among them, reactive species are known to be the most powerful effector, and there is an additional generation mechanism in PAL compared with direct application of plasma.

**Figure 1:**
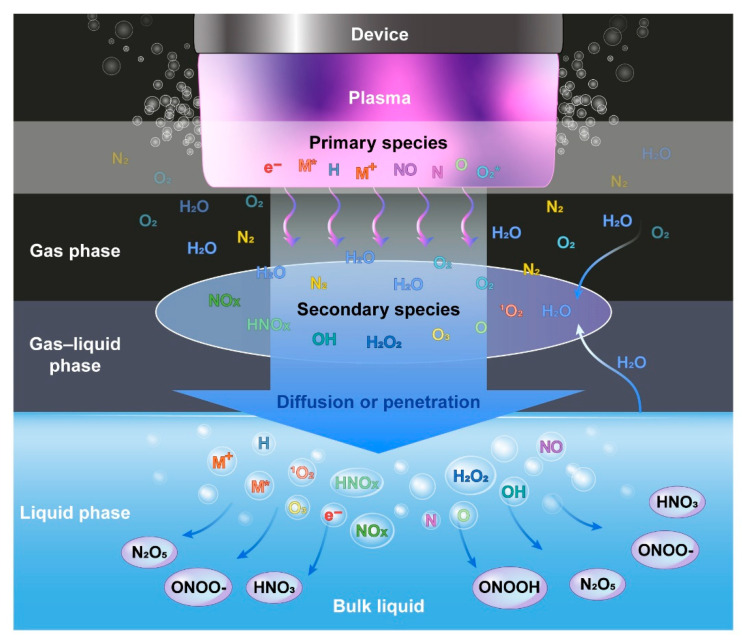
Schematic diagram of formation of reactive species in PAL [21,23,24].

The reactive species seen in PAL is a mixture of oxygen species (O, O_3_, ^1^O_2_, •OH, H_2_O_2_, O2•^−^/•OOH) and nitrogen species (•NO, ONOO^−^ and OONOO^−^, NO_2_^−^, NO_3_^−^) [17]. There is an additional interface step with liquid in the radical generation process of PAL, so many efforts regarding the composition ratio, generation process, and source of reactive oxygen and nitrogen species (RONS) have been made to understand the interface step with liquid [18]. In summary, in PAL, RONS is produced in three phases. (1) Gas phase: A radical with a relatively short survival period (1.3–2.7 μs) as a primary species (•OH, •NO, •H, •O, •N) is generated [19]. Because of the high reactivity, they combine with other radicals or with surrounding gases to form secondary species (H_2_O_2_, NO_2_, NO_3_, O_3_). (2) Gas–liquid interface: Primary and secondary species react with evaporated H_2_O from solution to form •OH, O, H, •NO, HNO_3_, O_3_, H_2_O_2_, HNO_2_, and so on [20]. (3) Liquid phase: The gaseous radicals dissolve in the solution, and some react with H_2_O to form ONOOH, N_2_O_5_, HNO_3_, ONOO^−^, and so on [21,22]. Figure 1 illustrates the formation of RONS during the generation of PAL.

There are numerous systems for generating PAL, and according to their properties, Bruggeman et al. classified them as below [23]:

(1) Direct liquid phase discharges;
(2) Gas phase plasmas producing reactivity in the liquid;
  - Without direct contact/electrical coupling with the liquid;
  - With direct contact/electrical coupling with the liquid (liquid electrode);
  - At the plasma liquid interface (surface discharges)
(3) Multiphase plasmas
  - Gas phase plasmas with dispersed liquid phase (aerosols);
  - Gas phase plasmas dispersed in the gas phase (bubbles) in liquid

In conjunction with PAL generation, there are two ways to treat targets with PALs. The direct type of treatment is the plasma treats liquid when the liquid contains the target. This method usually permits a flux of various uncharged species as well as UV radiation from the plasma to the surface of the biological target. Moreover, the important feature of this type is the existence of a significant flux of negatively and positively charged species reaching the surface of the target. The efficacy of this method is in close relation to short-living species, such as singlet oxygen ^1^O_2_, and radicals, such as •OH, and atomic radicals, which are directly formed by the plasma [25,26]. The second type of treatment is to make PAL and then contact the PAL with the target, so-called indirect treatment [17]. In this case, the effects involve changes in the solution’s pH and conductivity, which affect the reactivity of chemical species, or postplasma reactions whereby degradation continues for a long time after the plasma treatment. For this method, the formation of relatively long-living species, such as H_2_O_2_ and O_3_, as well as secondary radicals generated in the liquid phase, is the most essential reactive species [25,26].

The conditions that can affect the RONS composition during the PAL generation process are as follows depending on the phase. (1) gas phase: type of plasma power supply, type of carrier gas, electrode configuration, applied voltage, voltage polarity, treatment time, and gas flow; (2) gas–liquid interface: humidity, vapor of liquid, size of interaction area, and distance between gas and solution; and (3) liquid phase: volume of solution, type of solution, distance between electrode and liquid surface, composition of solution, and pulse duration [15,22,27]. Gorbanev et al. confirmed the ROS composition ratio according to different environments [25,26]. It was confirmed that H_2_O_2_ is affected by the humidity of the feed gas, •OH is affected by the distance between the jet and liquid sample, H is generated on the surface of the liquid sample, and O_3_, O_2_^−^, and O are affected by O_2_ in the feeding gas. RNS was also found to be affected by humidity [28].

The composition ratio of RONS may vary depending on the generation system of PAL and the aforementioned parameters. Royintarat et al. showed the difference in the characteristics of PAL caused by different generation systems. They treated deionized water with two generation systems, plasma jet and dielectric barrier discharge (DBD). The results of optical emission spectroscopy were different between PAL with plasma jet and PAL with DBD. When mixed gas with Ar and O_2_ was supplied, a high intensity at λ = 777 nm was found in PAL with plasma jet but not with DBD. In bacteria reduction efficiency, PAL from plasma jet reduced *Staphylococcus aureus* and *Enterococcus coli* more than PAL from DBD [29]. Gorbanev et al. found that the concentration of •OH in the liquid was higher when the distance between the plasma nozzle and the liquid was 10 mm than when the distance was 4 mm [25]. Considering these results, the generation system and the parameters should be selected according to the purpose. The materials used for PAL creation per the literature are summarized in Table 1.

## 3. Application of PAL in Medicine

### 3.1. Sterilization and Disinfection

PAL was found to be effective against bacterium, fungus, and virus [36,37,38] that it is quite predictable that PAL would work. Since plasma can react with culture media in vitro and fluid (moist or wet due to exudation of wound) of surrounding tissues in vivo, there would be an interaction between plasma and liquid, which could have affected the results [14].

In early PAL research, it was thought that acidification during PAL generation would cause inactivation of microorganisms [39,40,41]. However, Oehmigen et al. confirmed that inactivation was impossible with acidic conditions only, and RONS is an important factor in microorganism inactivation [42]. In subsequent studies, experiments were conducted to confirm which reactive species caused the inactivation effect. Shainsky et al. reported a high concentration of hydrogen peroxide in PAL and claimed hydrogen peroxide and superoxide attributed to strong oxidizing properties [43]. Satoh et al. showed that PAL using O_2_ plasma resulted in higher inactivation than N_2_ and air plasma, and that it was the main effector of O_3_ when O_2_ plasma was used [44]. Korshunov et al. claimed that hydroperoxyl radical (HOO•) would cause inactivation because superoxide could not pass through the cell wall [45]. In addition to ROS, RNS (NO_2_, NO_3_, ONOO^−^) was also considered to be the main effector [46,47,48,49]. The mechanism of action of PAL regarding bacterial inactivation is different between Gram-negative and Gram-positive bacteria. In Gram-negative bacteria, RONS induced lipid peroxidation of the wall, which is a key player in cell death, including apoptosis, autophagy, and ferroptosis [50]. Lipid peroxidation produces a malondialdehyde (MDA), which interacts with nucleic acids and induces mutations [51,52]. In Gram-positive bacteria, the impact of RONS on intracellular components rather than lipid peroxidation is thought to be the main cell death mechanism. Han et al. reported the absorbance at 260 nm, which indicates the release of intracellular DNA and protein increases in *E. coli*, not in *S. aureus* [53]. UV and shock wave generated during PAL generation are also thought to be involved in inactivation [54,55].

Research related to microorganism inactivation has recently led to the study of the effect of PAL on biofilm. According to the International Union of Pure and Applied Chemistry, biofilm is defined as “aggregate of microorganisms in which cells that are frequently embedded within a self-produced matrix of extracellular polymeric substance (EPS) adhere to each other and/or to a surface” [56]. Microorganisms have several defense mechanisms against antimicrobial agents by forming biofilms [57]. Thus, they are one of the causes of infectious status, such as contamination of medical equipment or sepsis in the human body [58,59,60]. Due to various defense mechanisms, biofilms after 48 h become resistant to various drugs and are difficult to remove [61]. It has been reported that PAL is effective in the removal of biofilms. Chen et al. produced PAL by treating deionized water, saline, and citrate solution with a plasma jet using helium as a supplier gas for 60 min. As a result of contacting a biofilm made by culturing *E. coli* or *S. aureus* with PAL for 24 h, 99.5% of *S. aureus* was removed after 3 h, all bacterial cells disappeared after 16 h, 99.5% of *E. coli* disappeared after 1 h contact, and all bacteria died after 3 h [62]. Tan et al. observed antibiofilm activity using PAL in which distilled water was exposed to a plasma jet with air for 5 min. The pipe was inoculated with nalidixic-acid-resistant *Enterobacter aerogenes* solution for 48 h (24 h of circulation plus 24 h of incubation) to form a biofilm, and cleaning with PAL was at a speed of 0.11 m/s. The biofilm of the inner surface decreased compared with distilled water, and bacterial concentration in the solution used for cleaning also decreased [63].

It is difficult to access the internal spaces of complex tools. A GI endoscope, which has two lumens for biopsy and air/water irrigation, is exposed to many microorganisms, and sterilization is important. Balan et al. used PAL in which distilled water was exposed to gliding arc discharge with air for 10 min for the reprocessing of a duodenoscopy [64]. After 30 min of exposure to PAL, culture was performed outside the lumen of the device, and as a result, all strains showed absence of growth in culture media. The surface of the device was not damaged. As another example of applying PAL to the lumen of a tool, PAL is used to remove biofilm in the lumen of an endotracheal tube [65]. Antibiofilm efficacy was measured by nebulizing 4 mL of solution (deionized water, phosphate-buffered saline, N-acetylcysteine) exposed to DBD for 3 min at a rate of 6 slm (standard liter per minute). As a result, all three types of solutions showed significant effects. As another example of vaporized application, Wong et al. treated deionized water with plasma and then made an aerosol with acoustic waves to see the antimicrobial activity, which showed a 95% reduction in 30 min of treatment [66]. These experiments show that PAL has an antimicrobial effect not only in the bulk liquid state, but also in vaporized or aerosol form.

The central venous catheter is a problematic instrument due to biofilm formation. A central venous catheter is inserted for patients in need of high-volume replacement. Antibiotic lock is recommended because biofilms in the catheter lumen can form during prolonged use, which can cause bloodstream infection [67,68,69]. Bhatt et al. reported that when the catheter where biofilm was formed in the lumen was locked with PAL and M-EDTA-25E (3 mg/mL minocycline hydrochloride and 30 mg/mL EDTA mixed in 25% ethanol) for 60 min, PAL eradicated the biofilm and completely suppressed the regrowth of the biofilm. However, M-EDTA-25E showed residual bacterial population and microbial recovery [70].

PAL is a promising technology because it has sufficient antimicrobial effect and can be converted into an aerosol form when needed. In addition, it has recently been effective against COVID-19 [71], so it is thought that it can be sufficiently used as a sterilizer in public health.

### 3.2. Tissue Regeneration

The effect of cold plasma on wound healing was confirmed in a randomized clinical trial conducted with the direct application of plasma in diabetic foot ulcers [72,73,74]. Based on the studies conducted with the direct application of plasma, the wound healing mechanism can be summarized into seven types [75,76]: (1) inactivated pathogenic bacteria, (2) promoting re-epithelization and accelerating the wound closure, (3) activating wound-healing-relevant cytokines and growth factors in fibroblasts and keratinocytes, (4) inducing neovascularization, (5) activating fibroblasts inducing the rearrangement of the actin cytoskeleton and promoting the matrix synthesis, (6) reducing inflammation by the recruitment of immune cells into the wound area, and (7) reducing inflammation by the activation of a body-protective mechanism.

There are two ways to apply PAL to wound healing. One is to apply PAL on the wound surface, and the other is to apply it under the wound. Xu et al. reported the effect of applying PAL on the wound surface. When a full-thickness wound with a diameter of 2 cm was made on mice and treated with PAL on days 0, 4, 7, and 10, the wound disappeared on day 17. It showed a significant difference when compared with the control group healed on the 23rd day. There was no difference between the control and PAL treatment groups in the blood biochemical indicator and histology of a major organ, so it was biologically safe [77]. Injecting PAL under the wound has also shown a wound healing effect. Won et al. made a vocal fold injury in a rabbit, and PAL was injected under the wound [78]. As a result, at 6 weeks after injection, there was no difference from the control, and the histologic collagen deposition was similar to normal. In another application method, Lee et al. observed wound healing in radiation-treated skin [32]. PAL treated with N_2_Ar for 3 min was mixed with silk fibrin to form a hydrogel. After performing full-thick flap elevation of the radiation-treated skin, the hydrogel was placed subcutaneously, and the skin flap was returned to its original position. As a result, the plasma-treated silk-fibrin hydrogel group showed better wound healing than the silk-fibrin hydrogel-treated group and the untreated group. This study showed a good example of increasing utilization by adding additives to PAL. There is a study applying PAL in the form of hydrogel to vitiligo, an autoimmune disease. Following deionized water treatment with plasma, hydroxyethyl cellulose was mixed and treated once every other day in mice and patients. Vitiligo-related depigmentation, CXCL10, and dendritic cell and T cell (CD3+, CD8+) infiltration were reduced in mice, and the same results were obtained in a clinical trial involving 20 patients [34].

PAL has the potential for deep tissue regeneration and skin healing. Park et al. treated a petri dish containing myoblast cells with media by N_2_Ar gas plasma. As a result of Western blot, it was found that muscle differentiation markers such as MyHC and myogenin increased in the plasma-treated group [79]. PAL was applied for the healing of another deep tissue, astrocyte (one of the neuroglia of the central nervous system). Sardella et al. processed liquid media with plasma using N_2_, Air, and O_2_ as feeding gas, and then scratch-induced wound healing assay was performed with astrocytes. It was confirmed that proliferation and migration were increased in all of N_2_, air, and O_2_ [80]. In addition, plasma not only is involved in skin reepithelization, but also has shown effects on functional aspects, such as hair thickness and regrowth [81,82]. Based on these results, a clinical trial is being conducted to apply PAL to androgenic alopecia [83].

### 3.3. Cancer Treatment

ROS can stimulate cell proliferation, but it causes cell death when the amount of ROS within the cell exceeds a certain level. When ROS flows into cancer cells with elevated intracellular ROS levels due to metabolic abnormalities, the intracellular ROS level of cancer cell reaches the toxicity threshold more easily than in normal cells [84]. Due to this characteristic of cancer cells, the plasma has selectivity (kills cancer and has no effect on normal cells). Plasma selectivity has been identified in carcinomas of various organs, including brain [85], skin [86], breast [87], head and neck [88], blood [89], ovary [90], gastrointestinal [91,92], bone [93], and lung [94]. Due to this selectivity, plasma is considered to be a very novel tool for cancer therapy and has received a lot of attention [95]. In brief, looking into the mechanism of anticancer action, (1) secondary ^1^O_2_ is generated by the biochemical composition (containing NOX1, catalase, and SOD) of the tumor cell membrane; (2) secondary ^1^O_2_-mediated catalase inactivation occurs; (3) H_2_O_2_ flows into the cell through aquaporins; (4) intracellular ROS signaling is induced; and (5) mitochondrial apoptosis signaling occurs, resulting in cancer cell death [96]. Although H_2_O_2_ is considered to be the main effector, a synergistic effect of H_2_O_2_ together with NO*_x_* has been observed in several studies [97,98], and this synergy is thought to lead to plasma selectivity [99]. Today, the application of plasma to anticancer treatment is beyond the proof-of-concept research stage. Short- and long-term experience of treatment with the direct application of plasma has been shared in head and neck cancer. Metelmann et al. reported partial remission of tumor cell after treatment with the direct application of plasma for 3 weeks (three single treatments within 1 week, followed by an intermittence of 1 week without plasma exposure), and the median survival time of patients was 7.5 months [100,101]. Clinical trials are in progress in cervical cancer [102,103]. It is expected to be applied to actual treatment in the near future.

Cancer existing in a deep tissue can be treated with PAL. According to Partecke et al., apoptosis level caused by the direct application of plasma is affected at a depth of 48.8 ± 12.3 μm [104], so if the cancer is located in the deep tissue, the anticancer effect of plasma through direct application will be limited. Additionally, diffuse peritoneal metastasis may occur in cancers of the abdominal cavity, which generally has a large surface area. When the cancer is present in the deep tissue or peritoneal cavity, PAL can be approached by intratumoral or intraperitoneal injection.

Kim et al. confirmed tumor size decrease following intratumoral injection of PAL to a subcutaneous head and neck cancer mouse model [105]. They made PAL as PBS treated with a plasma jet using helium and oxygen as carrier gases for 15 min and performed daily intratumoral PAL injection of 200 μL in a syngeneic mouse model for 6 days and in a xenograft model for 10 days. In both models, tumor volume and tumor weight were significantly reduced, and mitochondrial E3 ubiquitin protein ligase 1 (MUL1) was thought to be involved in this process.

The German group confirmed that the tumor burden was reduced in mice with peritoneal carcinomatosis by intraperitoneal injection of PAL [91,106]. Liedtke et al. created an in vivo model by intraperitoneal injection of the murine pancreatic cancer cell line 6606PDA into mice. From the 7th day, intraperitoneal injection of 1 mL of PAL (Dulbecco’s Modified Eagle’s Medium (DMEM) treated with argon plasma jet for 10 min) was performed every day. After 2 weeks of treatment, the tumor size increase in the plasma-treated group was significantly lower than that in the control group, and after 3 weeks of treatment, the total tumor mass in the treated group showed a significant decrease by 31.3% in the control group [91]. Additionally, for survival analysis, they performed euthanasia on day 70 after 35 injections up to day 42 and observed that the median survival was 61 days in the plasma-treated group, which was significantly longer than the 52 days in the control group [91]. Freund et al. reported the results after injecting CT26 colorectal cancer cells into the peritoneum of the mice, where 300 μL of 0.9% sodium chloride solution treated with plasma jet for 60 min was intraperitoneally injected five times every other day from day 2. They found that the tumor mass was significantly reduced by one-third in the plasma-treated group compared with the control group [106].

The anticancer effect of PAL can be sustained after storing. According to Adachi et al. and Judée et al., the anticancer effect was still maintained when PAL was made with DMEM and stored at −80 °C for 7 days [107,108]. However, when PAL was stored at 4 °C, Judée found that PAL was effective after storing, which was not found in the study of Adachi. There may be several reasons for this difference, one of which is the type of liquid used. Yan et al. checked the H_2_O_2_ concentration after storing DMEM and PBS treated with plasma for 26 h at 8 and 22 °C. The concentration of H_2_O_2_ was decreased in DMEM but not in PBS. In addition, even the same type of solution showed different anticancer effects depending on the composition of amino acids. After adding 15 specific amino acid solutions to DMEM to make modified DMEM and storing it at 22 °C for 26 h, the H_2_O_2_ concentration was decreased noticeably in modified DMEM with cysteine, methionine, and phenylalanine. After daily storage at 8 °C for 3 days, cell viability was lower in DMEM without cysteine and methionine compared with DMEM with both. To compensate for this, when 3-nitro-L-tyrosine was mixed with DMEM, the concentration of H_2_O_2_ was increased, and cell viability was lower compared with DMEM without mixing when stored at 8 °C for 3 days [109].

There is a study using hydrogel for cancer therapy just as hydrogel was used for wound healing. Zang et al. reported that after surgical removal of subcutaneously implanted cancer in mice, a plasma-treated thermosensitive biogel ((poly-DL-lactide)-(poly-ethylene glycol)-(poly-DL-lactide) PLEL) was inserted into the place where cancer was removed. The recurrence in the group with the plasma-treated biogel was significantly lower than that in the surgery-only group, and there were no abnormalities in organ histology and blood cell count in terms of safety [33].

### 3.4. Antiangiogenesis

Induction of neovascularization through nitric oxide is well known as one of the wound healing mechanisms of plasma [110,111,112]. According to Ye et al., PAL has an antiangiogenesis effect. In the case of wet-type age-related macular degeneration, choroidal neovascularization (CNV) invades the retina and causes blindness. Intravitreal injection of antivascular endothelial growth factor (VEGF) agent is currently the standard therapy. Ye et al. confirmed that intravitreal injection of PAL in a wet-type AMD mouse model did not affect pre-existing retinal vessels, but caused apoptosis of CNV-producing endothelial cells [113]. This result supports the possible application of PAL to the existing antiangiogenesis therapy, such as cancer or orbital disease.

### 3.5. Plasma Activation of Various Solutions

One of the strengths of PAL is that it can be made with various liquids suitable for the purpose of use. Sodium hypochlorite (NaOCl) is a commonly used disinfectant, often at a concentration of 200 ppm. An et al. made PAL with 100 ppm NaOCl and applied it to a biofilm made by culturing *E. coli* O157:H7 for 5 days. Plasma-treated 100 ppm NaOCl solution showed a >3 log CFU/cm^2^ high bacterial reduction compared with 200 ppm NaOCl solution not treated with plasma, suggesting that using plasma can show a better effect with a small amount of NaOCl [114]. Regarding the media used for cell culture, PAL can be made using various media, as well as PBS and DMEM. Wende et al. compared cell viability by making PAL with Roswell Park Memorial Institute Medium 1640 (RPMI 1640) and Iscove’s modified Dulbecco’s Medium (IMDM), and HaCaT cell viability was maintained in IMDM after long-term treatment of plasma [35]. Biscop et al. made PAL with DMEM, RPMI 1640, Astrocyte Medium (AM), Bronchial Epithelial Growth Medium (BEGM), and Dermal Cell Basal Medium (DCBM) and compared the cell viability. The cell viability of a lung cancer cell line (A549) and a brain cancer cell line (U87) was decreased in DMEN and RPMI 1640 but not in AM and DCBM, which are advanced media for noncancerous cells [115].

There are two methods for making a hydrogel: mixing alginate after making PAL and the plasma treatment on a solution already mixed with alginate. The two methods may seem similar at first glance, but Labay et al. suggested that the latter contains more RONS. Labay et al. treated the alginate solution with plasma and confirmed that 0.5% alginate hydrosol treated with plasma produced significantly more RONS (NO_2_^−^, H_2_O_2_, short-lived species) than the control. In addition, cytotoxicity was not observed, and plasma treatment did not affect the physicochemical properties of alginate, so there was no problem in crosslinking using CaCl_2_ [116].

In addition to PAL based on water, PAL can be made based on oil. Zou et al. treated olive oil with the plasma jet for 7 h using helium and oxygen. Plasma-activated oil (PAO) has a higher peroxide value than traditional ozonated oil. PAO stored (SPAO) at a low temperature (2–8 °C, without sunlight) and a high temperature (25–35 °C, with sunlight) for 3 months had a higher peroxide value than traditional ozonated oil. In addition, both PAO and SPAO showed bactericidal activity against *E. coli* and MRSA, and the wound closure rate of both in the in vivo wound healing model was significantly higher than that in the control group [117]. Xu et al. made PAO with sunflower oil and applied it to infected wound healing. They confirmed that it was effective against MRSA, *Micrococcus luteus*, *E. coli*, and *P. aeruginosa* through an in vitro experiment, and confirmed that the wound area was significantly reduced compared with the control when treated with PAO in the in vivo infected wound model [30]. Zou et al. and Xu et al. claimed that carboxylic acid is produced when the C=C bond, which does not exist in water, is broken by plasma and reactive species from the cleavage exhibit acidification and antimicrobial activity.

### 3.6. Safety

Confirmation of safety for the medical use of plasma is of utmost importance. When PAL was applied to wound healing in a mouse model, there was no obvious abnormal change in hematoxylin and eosin staining of major organs (heart liver, spleen, lung, and kidney), and there was no difference in blood biochemical indicators [77]. In the cancer mouse model, there was no difference in blood leukocyte and lymphocyte composition, count of erythrocytes and thrombocytes, and level of cytokine even during peritoneal injection [91]. Abnormality in blood leukocyte component and liver function was not found even during intratumoral injection or in the renal function using urine [118]. Xu et al. confirmed acute toxicity and systemic toxicity by conducting oral lavage with PAL in immune-deficient mice [119]. There was no acute toxicity, and there were no abnormalities in organ weight, organ coefficient (organ weight/body weight × 100%), histology of organ slice, blood routine test, liver function test, kidney function test, and glucose and lipid metabolism. Xu et al. made PAL using three types of solution (0.9% NaCl, PBS, medium (10% fetal bovine serum and 1% penicillin–streptomycin solution)) in rabbit and injected it intramedullarily to evaluate systemic toxicity [120]. There was no acute toxicity in all three types, organ coefficient (heart, liver, spleen, lung, kidney), macroscopic and microscopic lesion, and blood test (cell count, biochemical indicators of liver and kidney function, electrolyte levels, myocardial enzymes, antioxidant levels, glucose metabolism, and lipid metabolism). The previous results are for short-term safety. A study on long-term safety is needed for the implementation of PAL to real practice.

## 4. Future

Plasma started with the decontamination of a surface and media with atmospheric pressure plasma in the United States in 1996, and now, many studies are being conducted that cannot be compared with those in the early days [121,122]. In medicine, plasma has received a lot of attention as a novel tool, and the field of plasma medicine was created, and PAL is also being discussed as an aqueous plasma pharmacy as a field within plasma medicine [16,39]. In the area of PAL, in addition to the wound healing, disinfection, and cancer therapy mentioned above, active research is being conducted on making plasma-activated oil or hydrogel [116,117], combined therapy with other treatments [123], and interaction with drugs [124].

The direct application of plasma was approved in the US in 2008 and is being used in actual clinical trials in Europe and Japan [122]. In Germany, general requirements for plasma sources in medicine was enacted in 2014, and good clinical practice was established for clinical trials, and a guideline project was started for medical use in 2018 [125]. However, in the PAL field, the movement for standardization has not been made due to some unclear areas, such as the use of various generation devices and feeding gases [95], and different effects depending on the type of liquid and cell line [126]. Therefore, it is necessary to solve the following parts: (1) a thorough understanding of the plasma generation (method of discharge, scale, exposure time, electrode corrosion, etc.), (2) interaction between plasma and liquid (solution composition, analytical methods optimization, etc.), and (3) bioapplication (dosage, mode of action, route of administration, safety, regulatory) [14,15,16].

PAL and the direct application of plasma have complementary characteristics. If PAL can be used in clinical practice, the quality of treatment can be improved by selecting between two applications according to the characteristics and region of the disease.

## Figures and Tables

**Table 1:** Possible options for PAL [16,23,30,31,32,33,34,35].

Generation System	Solution	Additive
Plasma jet Dielectric barrier discharge Microwave	Tap water Deionized water Distilled water DMEM PBS 0.9% NaCl Ringer’s lactate solution Oil	Hydroxyethyl cellulose Fibrin Alginate PLEL
